# Longitudinally and circumferentially directed movements of the left ventricle studied by cardiovascular magnetic resonance phase contrast velocity mapping

**DOI:** 10.1186/1532-429X-12-48

**Published:** 2010-08-17

**Authors:** Ion Codreanu, Matthew D Robson, Stephen J Golding, Bernd A Jung, Kieran Clarke, Cameron J Holloway

**Affiliations:** 1 Department of Physiology, Anatomy and Genetics, University of Oxford, Oxford, UK; 2 University of Oxford Centre for Clinical Magnetic Resonance Research, Oxford, UK; 3 MRI Centre, John Radcliffe Hospital, University of Oxford, Oxford, UK; 4 Department of Diagnostic Radiology, Medical Physics, University Hospital, Freiburg, Germany

## Abstract

**Objective:**

Using high resolution cardiovascular magnetic resonance (CMR), we aimed to detect new details of left ventricular (LV) systolic and diastolic function, to explain the twisting and longitudinal movements of the left ventricle.

**Methods:**

Using CMR phase contrast velocity mapping (also called Tissue Phase Mapping) regional wall motion patterns and longitudinally and circumferentially directed movements of the left ventricle were studied using a high temporal resolution technique in healthy male subjects (n = 14, age 23 ± 3 years).

**Results:**

Previously undescribed systolic and diastolic motion patterns were obtained for left ventricular segments (based on the AHA segmental) and for basal, mid and apical segments. The summation of segmental motion results in a complex pattern of ventricular twisting and longitudinal motion in the normal human heart which underlies systolic and diastolic function. As viewed from the apex, the entire LV initially rotates in a counter-clockwise direction at the beginning of ventricular systole, followed by opposing clockwise rotation of the base and counter-clockwise rotation at the apex, resulting in ventricular torsion. Simultaneously, as the entire LV moves in an apical direction during systole, the base and apex move towards each other, with little net apical displacement. The reverse of these motion patterns occur in diastole.

**Conclusion:**

Left ventricular function may be a consequence of the relative orientations and moments of torque of the sub-epicardial relative to the sub-endocardial myocyte layers, with influence from tethering of the heart to adjacent structures and the directional forces associated with blood flow. Understanding the complex mechanics of the left ventricle is vital to enable these techniques to be used for the evaluation of cardiac pathology.

## Background

Left ventricular (LV) function is geometrically and mechanically complex. Advances in cardiac imaging techniques have accompanied ongoing efforts to define the mechanisms of three dimensional ventricular motion [1-7]. Current theories explaining LV motion are controversial. The "myocardial band model" divides the myocardium into two distinct helicoids [6,7], but fails to explain the mechanisms of myocardial contraction after repolarization and ventricular motion in diastole. Additionally, the embryological development of the heart, and the failure of anatomists to separate "bands" on anatomical dissection, have further challenged this theory [8-12].

Cardiovascular magnetic resonance (CMR) has allowed detailed evaluation of LV wall motion throughout the cardiac cycle, using myocardial velocity encoding techniques [13,14]. Here, we have used results from CMR phase contrast velocity mapping with high temporal resolution, to characterise longitudinal and rotational movements of the left ventricle in healthy human subjects.

## Methods

CMR scans were performed on fourteen healthy male volunteers (age 23 ± 3 years). All subjects were non-smokers, with no history of cardiovascular disease. The study was conducted according to the principles of the Declaration of Helsinki and was approved by a local Oxfordshire Clinical Research Ethics Committee. Each subject provided written informed consent.

### CMR

CMR scans were performed using a 1.5 Tesla Siemens Sonata clinical scanner. Pilot images, followed by horizontal and vertical long axis cine images were acquired using an SSFP pulse sequence. Cine images for navigator gated high temporal resolution phase contrast velocity mapping were acquired using a *black blood segmented k-space *spoiled gradient echo sequence (TR = 13.8 ms, TE = 5.0 ms, flip angle = 15°, bandwidth = 650 Hz/pixel, FOV = 400 × 300 mm, matrix = 256 × 96) [13,14].

To obtain velocity measurements, three equidistant short-axis slices along the left ventricle were evaluated using 3-directionally encoded, time resolved phase contrast velocity maps. Velocity encoding was performed by including a phase image with no velocity encoding followed by images with a bipolar gradient in read, phase or slice direction after each RF pulse to the otherwise identical sequence (venc in-plane = 20 cm/s, venc through-plane = 30 cm/s). Acquisition was prospectively gated, typically covering between 80 to 90% of the cardiac cycle, meaning the contribution of atrial contraction on left ventricular motion was not possible to assess. Post-processing was performed in the standard fashion of subtracting the phase from the image with no velocity encoding, followed by conversion of the phase data into velocity maps.

The basal slice was positioned parallel to the base of the heart, distal to the LV outflow tract. Basal, midventricular and apical slices were positioned 15 to 20 mm apart, depending on the heart size. With cardiac and respiratory gating, each short axis acquisition took approximately 3-5 minutes, with an average of 60-70 phases per cardiac cycle. Cardiac phases were determined, with the end systole defined as the smallest LV cavity.

Analysis was performed using customized software (Matlab, version 6.5; Mathworks, Natick, Mass), where the left ventricle was divided into 16 segments (six basal, six middle and four apical) according to the American Heart Association model [15]. Ventricular velocities and regional wall motion patterns were determined for all individual segments. The myocardial velocity results, or time varying vectors derived from all 3 directional components of velocity, have been subdivided into "longitudinal" and "circumferential" components (from one through-plane component, and two in-plane velocity datasets, respectively). All myocardial velocities are expressed as cm/s. Circumferential velocities are described as clockwise or counter-clockwise, as viewed from the ventricular apex.

## Results

### Rotational motion

**Figure 1:**
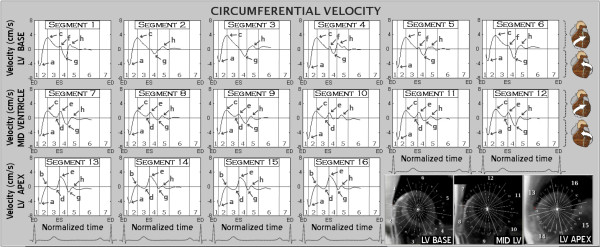
**Circumferential velocity graphs for LV segments according to the AHA segment model (segments 1-6 are basal, 7-12 midventricular and 13-16 apical)**. The graphs represent the average values of all volunteers. Positive values show clockwise rotation as viewed from the apex, whilst negative values represent counter-clockwise rotation. Phases of the cardiac cycle: 1 - isovolumetric contraction, 2 - rapid ejection, 3 - reduced ejection, 4 - isovolumetric relaxation, 5 - rapid filling, 6 - diastasis, 7 - atrial systole, ED - end diastole, ES - end systole.

**Figure 2:**
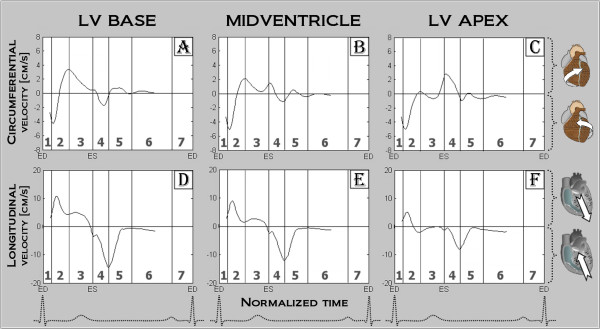
**Average longitudinal and circumferential velocities for the left ventricular base, mid and apex**. The entire LV rotates counter clockwise (as viewed from the apex), followed by a series of clockwise and counter clockwise motion patterns, leading to opposing directions of apical and basal motion during a cardiac cycle. In all slices, longitudinal motion of the heart is towards the apex during systole and towards the base during diastole.

At the beginning of systole, all LV segments rotated in a counter-clockwise direction, as viewed from the apex, reaching peak velocities at the beginning of rapid ejection (Figure 1, arrow *a*). By the middle of the rapid ejection phase, the basal and mid-ventricular segments (segments 1-6 and 7-12, respectively, Figure 1) reversed their rotational motion, reaching peak clockwise velocities by the end of this phase (Figure 1, arrow *c*). The apical segments, however, continued their counter-clockwise rotation until ventricular repolarization occurred (segments 13-16, wave *d*, Figure 1). Cardiac repolarization was associated with a sudden change in the direction of movement for apical and mid-ventricular segments, which started rotating in a clockwise direction (Figure 1, arrow *e*). In most basal segments, a brief increase in clockwise motion occurred at the beginning of diastole (Figure 1, arrow *f*). By the end of isovolumetric relaxation, the entire ventricle was rotating in a counter-clockwise direction (Figure 1, arrow *g*), with peak rotational velocities reached from the LV base towards the apex. This wave of recoil motion was particularly prominent in anterior (1, 7) and anterolateral (6, 12) segments. Subsequently, the amplitude of the rotational movement decreased with little circumferential movement during diastasis. Average motion for basal, mid and apical slices are provided in Figure 2.

**Figure 3:**
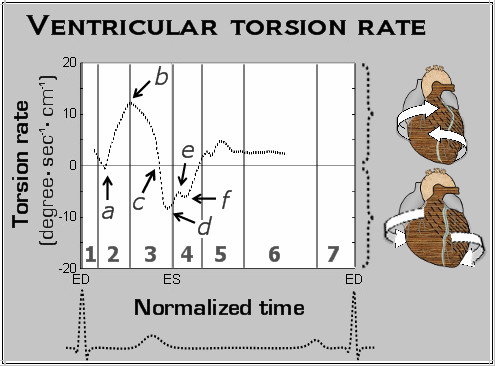
**Global LV torsion rate during a cardiac cycle**. Ventricular torsion reflects the base to apex gradient resulting from the twisting motion of the ventricle, whilst the torsion rate reflects the speed at which this twisting motion occurs. The entire ventricle rotates counterclockwise at the beginning of systole, resulting in little gradient between the LV base and apex, with the torsion rate close to zero (*a*). Subsequently, as the LV base rotates in a clockwise direction, the ventricular torsion rate increases, reaching its peak value at the end of rapid ejection (*b*). Then, as the clockwise velocities of the ventricular base fall during the phase of reduced ejection, the ventricular torsion rate similarly declines. Repolarization was followed by a sudden onset of ventricular untwisting, reflected in a negative ventricular torsion rate (*c*). Subsequent smaller negative waves of the ventricular torsion rate (*d, e, f*) likely correspond to slightly different peaks of ventricular untwisting at the LV base and apex.

The variation in global ventricular torsion rate, which reflects the speed of ventricular twisting motions or the gradient between apical and basal motion, is displayed in Figure 3. There was little gradient between the LV base and apex at the beginning of systole, with torsion rates close to zero (Figure 3, arrow *a*). Subsequently, the torsion rate progressively increased, peaking at the end of rapid ejection (Figure 3, arrow b), followed by a gradual decline during the phase of reduced ejection. Repolarization was followed by a sudden change in the direction of ventricular twisting, reflected in a negative ventricular torsion rate (Figure 3, arrow *c*) and subsequently in negative waves on the ventricular torsion rate graph (Figure 3, arrows *d, e, f*).

### Longitudinal motion

**Figure 4:**
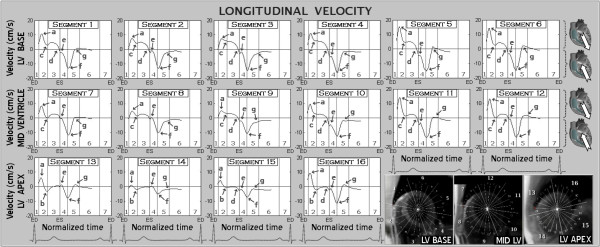
**Longitudinal velocity graphs for LV segments according to the AHA segment model (segments 1-6 are basal, 7-12 midventricular and 13-16 apical)**. The graphs represent the average values of all volunteers. Positive values show downward movement along longitudinal axis (towards the ventricular apex), while negative values reflect upward displacement. Waves *a *to *g *- see text for ventricular motion description, 1 - isovolumetric contraction, 2 - rapid ejection, 3 - reduced ejection, 4 - isovolumetric relaxation, 5 - rapid filling, 6 - diastasis, 7 - atrial systole, ED - end diastole, ES - end systole.

At the beginning of systole, all segments moved downwards (towards the LV apex) along the LV longitudinal axis, reaching peak velocities during the first half of rapid ejection (Figure 4, arrow *a*). By the end of the rapid ejection phase, the velocity of this downward movement decreased to a plateau in basal and mid-ventricular segments (Figure 4, arrow *c*), whilst at the apex it reached negative values, indicating the apex was moving towards the base of the heart (Figure 4, arrow *b*). Overall, there was little apical displacement during systole. Repolarization was followed by a steep deceleration in downward displacement (Figure 4, arrow *d*) with a change in movement direction before the end of systole. Subsequently, a fast recoil motion in an opposite direction occurred in all segments. This upward movement along the longitudinal axis continued during isovolumetric relaxation, reaching peak velocities by the end of this phase (Figure 4, arrow *f*). There was a brief deceleration in the upward recoil displacement at the beginning of diastole (Figure 4, arrow *e*). During the phase of rapid ventricular filling, the longitudinal velocity abruptly fell in all segments (Figure 4, *g*) with little subsequent motion along the longitudinal axis.

**Figure 5:**
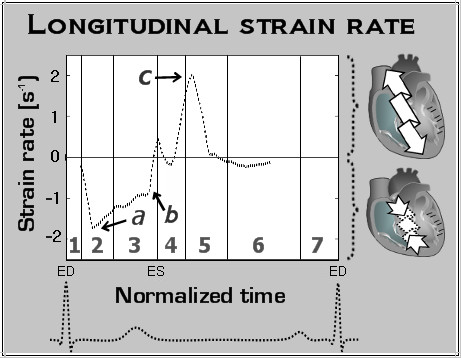
**LV longitudinal strain rate during a cardiac cycle**. Positive strain rate values correspond to LV lenthening, where negative values indicate LV shortening. This figure demonstrates a progressive rate of longitudinal shortening with the onset of rapid ejection (phase 2), followed by a relatively constant shorthening rate throughout the rest of the rapid and reduced ejection phases. Ventricular repolarizatioin was followed by a sudden drop in longitudinal shortening with a subsequent peak of longitudinal lenthening in early diastole. *a *- peak systolic longitudinal strain rate, *b *- sudden drop in longitudinal shortening rate after repolarization, *c *- peak diastolic longitudinal strain rate.

Both systolic and diastolic velocities were higher in basal segments, compared with midventricular and apical segments, showing greater displacement of the LV base in a longitudinal direction. The septal segments, which are a direct continuation of great vessels, showed lower values of peak systolic longitudinal motion (Figure 4, wave *a*, segments 2, 3, 8, 9, 14) compared to other segments from the same ventricular slice. In all segments diastolic velocities were considerably higher than corresponding systolic values, indicating rapid recoil motion shortly after the commencement of ventricular repolarization. Changes in overall longitudinal motion can also be appreciated using global longitudinal strain rate curves (Figure 5) showing the speed of myocardial deformation in a longitudinal axis. By convention, lengthening is represented as a positive strain value, while shortening is represented as a negative value. A progressive rate of longitudinal shortening was noted with the onset of rapid ejection (Figure 5, arrow *a*), followed by a relatively constant shorthening rate throughout the rest of the rapid and reduced ejection. Ventricular repolarization was followed by a sudden drop in longitudinal shortening (Figure 5, arrow *b*), with a subsequent peak of longitudinal lenthening in early diastole (Figure 5, arrow *c*).

## Discussion

These results demonstrate regional variations in circumferential and longitudinal motion within the left ventricular during systole and diastole. We believe this complex pattern of LV segmental motion, demonstrated using cardiac phase contrast velocity mapping, can be largely explained by the anatomical orientation of cardiomyocytes within the left ventricle and their attachments to adjacent structures.

**Figure 6:**
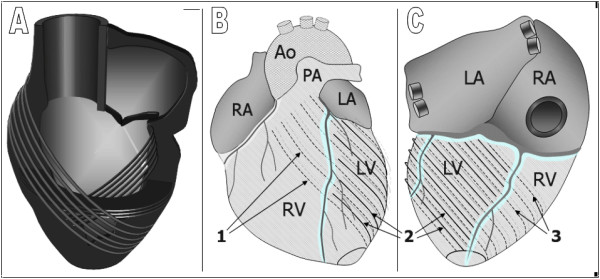
**Schematic representation of the spiraling orientation of cardiomyocytes, forming a three-dimensional mesh supported by the heart's fibrous matrix**. A - cross-section, B - anterior heart surface, C - posterior heart surface, 1 - outer surface aggregates extending from the left ventricle to the anterior heart surface, 2 - outer surface aggregates limited to the LV wall, 3 - outer surface aggregates extending to the posterior heart surface, Ao - aorta, PA - pulmonary artery, LA - left atrium, RA - right atrium, LV - left ventricle, RV - right ventricle.

LV rotation may be explained by the helical orientations of subepicardial and subendocardial myocytes forming a three-dimensional mesh supported by the fibrous matrix of the heart. As seen from the apex, these aggregates ascend in a clockwise fashion when traced from the apex to the base in the subendocardial zones and in a counter-clockwise direction at the outer surface [1,16-18]. Mathematical models have shown that this counter-directional helical arrangement of muscle fibers in the heart is important for equal redistribution of stresses and strain in the heart, which helps maintain stability whilst minimizing energy expenditure [19]. Studies on human hearts have shown the middle myocardial layer is approximately parallel to endocardial and epicardial surfaces, but oblique to those of other layers (Figure 6A). This perpendicular orientation theoretically provides additional strength to the myocardial wall during simultaneous contraction and may also enable contractile function of myocytes in different orientations to be combined. The outer-surface aggregates are longer and extend over a larger surface, thus have a greater radius of curvature and greater moment of torque. As such, they are likely to dominate the direction of circumferential motion. This is supported by work from Taber et al. who showed that, due to a larger moment arm, the outer layers have a mechanical advantage relative to the inner layers [20].

On the anterior aspect of the heart, a significant part of the outer-surface aggregates cross the interventricular sulcus, extending to the right ventricle, great vessels and fibrous structures. These aggregates (labelled as type 1 in Figure 6) have been described on manual dissection and recently using diffusion tensor magnetic resonance imaging [17,18]. Having the longest extension onto the anterior heart surface, they are likely to determine the dominant direction of initial LV rotation, causing the entire ventricle to rotate in a counter-clockwise direction, as viewed from the apex. We believe this explains the initial counter-clockwise movement of the left ventricle at the beginning of systole (Figure 1, wave *a*). Similarly, contraction of myocardial aggregates limited to the left ventricle (labelled as type 2 in Figure 6B and 6C) would result in LV torsion, i.e. in a clockwise rotation of the ventricular base (Figure 1, wave *c*) and a counter-clockwise motion of the apex, as observed in the velocity graphs. Since these aggregates are shorter, net motion only becomes apparent after the initial counter-clockwise rotation of the entire left ventricle, which is dominated by the longer (type 1) aggregates. The contraction of both type 1 and type 2 aggregates together may therefore explain the displacement of the ventricular apex anteriorly (Figure 6) and explain why the apex beat can be palpated on the anterior chest wall.

On the posterior aspect of the left ventricle, some of the outer surface aggregates also cross the interventricular sulcus, extending to the posterior surface of the right ventricle (labeled as type 3 in Figure 6C) [17,18]. The contraction of these aggregates may contribute to the clockwise rotation of the ventricular base during LV ejection, provided they end within the LV wall. Additionally, any of these aggregates extending to various support structures outside the left ventricle (great vessels, valvular rings and fibrous structures) may produce a biventricular counter-clockwise rotation during systole. Thus, contraction of outer surface aggregates, which extend to the anterior heart surface (type 1), may explain the initial counter-clockwise rotation of the entire left ventricle (Figure 1, wave *a*), whilst the simultaneous contraction of the outer surface aggregates limited to the left ventricle (type 2), explains the clockwise rotation of the LV base and the counter-clockwise movement of the apex (Figure 1, basal segments 1-6 vs. apical segments 13-16).

A key consideration for ventricular motion may be which parts of the heart (predominantly basal) are tethered in the mediastinum and thorax by large vascular and pericardial attachments, and which (more apical) are free to rotate. This is evident at the beginning of ventricular repolarization where there is a sudden change in movement direction occurring only at the apex (Figure 1, wave e in apical segments 13-16), where the net result for basal segments is a counter-clockwise rotation, superimposed on a brief clockwise rotation of the entire LV. A further consideration is the phasic, directional forces (and hence possible displacements) associated with the momentum changes of blood, particularly into the outflow tracts and great arteries in early systole. Slight torque may be associated with acceleration (and later deceleration) of blood into the helical curvatures of the great arteries, though this relationship is likely to be complex, so requires further evaluation.

Longitudinal motion of the LV during systole may be explained by contraction of the predominantly longitudinally oriented epi- and endocardial, trabecular and papillary myocytes. Repolarization leads to cessation of active myocardial contraction, although minimal blood flow still occurs at the end of reduced ejection due to the kinetic energy of the ejected blood [21]. There are two simultaneously occurring, and partly opposing components which likely contribute to longitudinal relaxation. Firstly, after repolarization, the LV base and apex move away from each other due to passive ventricular untwisting, resulting in an upward displacement of the base and a downward movement of the apex. Secondly, the potential importance of momentum changes, which are complex between atria, ventricles, outflow tracts and aortic arch should be considered. The results for basal motion would be consistent with elastic recoil, from these structures, pulling the ventricle back to its initial location after repolarization. The peak velocities of this upward longitudinal motion in diastole exceeding all other cardiac velocities during a cardiac cycle, strongly supports this "recoil" hypothesis.

Ventricular filling is likely to have multiple contributing factors. Firstly, a passive component, where repolarization leads to cessation of myocardial contraction and to ventricular wall relaxation, allowing blood to be expelled from the atria. Whether a suction component results from ventricular untwisting remains controversial [20,22]. We also believe there could be a mechanical, or recoil component, where the left ventricle is being pulled towards its initial location by the adjacent great vessels and mediastinum. This would lead to fast recoil motion against the filled left atrium and substantially increase the pressure difference across the mitral valve, contributing to ventricular filling.

The complex pattern of LV motion can be explained by the helical orientation of cardiomyocytes in the healthy heart. From these results we believe that left ventricular torsion is mainly a consequence of the relative orientations and moments of torque of the sub-epicardial relative to the sub-endocardial myocyte layers, with influence from tethering of the heart to the mediastinum and great vessels and the directional forces associated with blood flow. Whether assessment of these detailed systolic and diastolic parameters would assist in the early diagnosis of cardiac disease requires further evaluation.

## Competing interests

The authors declare that they have no competing interests.

## Authors' contributions

IC and CJH drafted the manuscript, MDR and BAJ participated in data interpretation and manuscript revision, SJG and KC were involved with study design and manuscript editing. All authors read and approved the final manuscript.

## References

[B1] Bovendeerd PH Arts T Huyghe JM van Campen DH Reneman RS Dependence of local left ventricular wall mechanics on myocardial fiber orientation: a model study J Biomech 1992 25 1129 1140 10.1016/0021-9290(92)90069-D 1400513

[B2] Bovendeerd PH Huyghe JM Arts T van Campen DH Reneman RS Influence of endocardial-epicardial crossover of muscle fibers on left ventricular wall mechanics J Biomech 1994 27 941 951 10.1016/0021-9290(94)90266-6 8063844

[B3] Buckberg GD Mahajan A Jung B Markl M Hennig J Ballester-Rodes M MRI myocardial motion and fiber tracking: a confirmation of knowledge from different imaging modalities Eur J Cardiothorac Surg 2006 29 Suppl 1 S165 177 10.1016/j.ejcts.2006.02.064 16569504

[B4] Burns AT McDonald IG Thomas JD Macisaac A Prior D Doin' the twist: new tools for an old concept of myocardial function Heart 2008 94 978 983 10.1136/hrt.2007.120410 18625792

[B5] Criscione JC Rodriguez F Miller DC The myocardial band: simplicity can be a weakness Eur J Cardiothorac Surg 2005 28 363 364 author reply 364-367 10.1016/j.ejcts.2005.04.015 15939612

[B6] Torrent-Guasp F Ballester M Buckberg GD Carreras F Flotats A Carrió I Ferreira A Samuels LE Narula J Spatial orientation of the ventricular muscle band: physiologic contribution and surgical implications J Thorac Cardiovasc Surg 2001 122 389 392 10.1067/mtc.2001.113745 11479518

[B7] Torrent-Guasp FF Whimster WF Redmann K A silicone rubber mould of the heart Technol Health Care 1997 5 13 20 9134615

[B8] Anderson RH Ho SY Redmann K Sanchez-Quintana D Lunkenheimer PP The anatomical arrangement of the myocardial cells making up the ventricular mass Eur J Cardiothorac Surg 2005 28 517 525 10.1016/j.ejcts.2005.06.043 16179192

[B9] Anderson RH Smerup M Sanchez-Quintana D Loukas M Lunkenheimer PP The three-dimensional arrangement of the myocytes in the ventricular walls Clin Anat 2009 22 64 76 10.1002/ca.20645 18567009

[B10] Henderson DJ Anderson RH The Development and Structure of the Ventricles in the Human Heart Pediatr Cardiol 2009 19225828 10.1007/s00246-009-9390-9

[B11] Manner J Ontogenetic development of the helical heart: concepts and facts Eur J Cardiothorac Surg 2006 29 Suppl 1 S69 74 10.1016/j.ejcts.2006.02.044 16563789

[B12] Sedmera D Form follows function: developmental and physiological view on ventricular myocardial architecture Eur J Cardiothorac Surg 2005 28 526 528 10.1016/j.ejcts.2005.07.001 16126399 PMC1389617

[B13] Jung B Foll D Bottler P Petersen S Hennig J Markl M Detailed analysis of myocardial motion in volunteers and patients using high-temporal-resolution MR tissue phase mapping J Magn Reson Imaging 2006 24 1033 1039 10.1002/jmri.20703 16947325

[B14] Jung B Markl M Foll D Hennig J Investigating myocardial motion by MRI using tissue phase mapping Eur J Cardiothorac Surg 2006 29 Suppl 1 S150 157 10.1016/j.ejcts.2006.02.066 16563784

[B15] Cerqueira MD Weissman NJ Dilsizian V Jacobs AK Kaul S Laskey WK Pennell DJ Rumberger JA Ryan T Verani MS American Heart Association Writing Group on Myocardial Segmentation and Registration for Cardiac Imaging. Standardized myocardial segmentation and nomenclature for tomographic imaging of the heart: a statement for healthcare professionals from the Cardiac Imaging Committee of the Council on Clinical Cardiology of the American Heart Association Circulation 2002 105 539 542 10.1161/hc0402.102975 11815441

[B16] Dorri F Niederer PF Redmann K Lunkenheimer PP Cryer CW Anderson RH An analysis of the spatial arrangement of the myocardial aggregates making up the wall of the left ventricle Eur J Cardiothorac Surg 2007 31 430 437 10.1016/j.ejcts.2006.11.040 17194601

[B17] Greenbaum RA Ho SY Gibson DG Becker AE Anderson RH Left ventricular fibre architecture in man Br Heart J 1981 45 248 263 10.1136/hrt.45.3.248 7008815 PMC482521

[B18] Rohmer D Sitek A Gullberg GT Reconstruction and visualization of fiber and laminar structure in the normal human heart from ex vivo diffusion tensor magnetic resonance imaging (DTMRI) data Invest Radiol 2007 42 777 789 10.1097/RLI.0b013e3181238330 18030201

[B19] Sengupta PP Korinek J Belohlavek M Narula J Vannan MA Jahangir A Khandheria BK Left ventricular structure and function: basic science for cardiac imaging J Am Coll Cardiol 2006 48 1988 2001 10.1016/j.jacc.2006.08.030 17112989

[B20] Taber LA Yang M Podszus WW Mechanics of ventricular torsion J Biomech 1996 29 745 752 10.1016/0021-9290(95)00129-8 9147971

[B21] Klabunde RE Cardiovascular Physiology Concepts 2005 2005 Lippincott Williams & Wilkins 65

[B22] Suga H Goto Y Igarashi Y Yamada O Nozawa T Yasumura Y Ventricular suction under zero source pressure for filling Am J Physiol 1986 25 1:H47 55 10.1152/ajpheart.1986.251.1.H47 3728698

